# HLA Class I Binding 9mer Peptides from Influenza A Virus Induce CD4^+^ T Cell Responses

**DOI:** 10.1371/journal.pone.0010533

**Published:** 2010-05-07

**Authors:** Mingjun Wang, Mette V. Larsen, Morten Nielsen, Mikkel Harndahl, Sune Justesen, Morten H. Dziegiel, Søren Buus, Sheila T. Tang, Ole Lund, Mogens H. Claesson

**Affiliations:** 1 Department of International Health, Immunology and Microbiology, Faculty of Heath Sciences, University of Copenhagen, Copenhagen, Denmark; 2 Center for Biological Sequence Analysis, Department of Systems Biology, Technical University of Denmark, Lyngby, Denmark; 3 H:S Blood Bank KI 2034, Copenhagen University Hospital, Copenhagen, Denmark; Federal University of São Paulo, Brazil

## Abstract

**Background:**

Identification of human leukocyte antigen class I (HLA-I) restricted cytotoxic T cell (CTL) epitopes from influenza virus is of importance for the development of new effective peptide-based vaccines.

**Methodology/Principal Findings:**

In the present work, bioinformatics was used to predict 9mer peptides derived from available influenza A viral proteins with binding affinity for at least one of the 12 HLA-I supertypes. The predicted peptides were then selected in a way that ensured maximal coverage of the available influenza A strains. One hundred and thirty one peptides were synthesized and their binding affinities for the HLA-I supertypes were measured in a biochemical assay. Influenza-specific T cell responses towards the peptides were quantified using IFNγ ELISPOT assays with peripheral blood mononuclear cells (PBMC) from adult healthy HLA-I typed donors as responder cells. Of the 131 peptides, 21 were found to induce T cell responses in 19 donors. In the ELISPOT assay, five peptides induced responses that could be totally blocked by the pan-specific anti-HLA-I antibody W6/32, whereas 15 peptides induced responses that could be completely blocked in the presence of the pan-specific anti-HLA class II (HLA-II) antibody IVA12. Blocking of HLA-II subtype reactivity revealed that 8 and 6 peptide responses were blocked by anti-HLA-DR and -DP antibodies, respectively. Peptide reactivity of PBMC depleted of CD4^+^ or CD8^+^ T cells prior to the ELISPOT culture revealed that effectors are either CD4^+^ (the majority of reactivities) or CD8^+^ T cells, never a mixture of these subsets. Three of the peptides, recognized by CD4^+^ T cells showed binding to recombinant DRA1*0101/DRB1*0401 or DRA1*0101/DRB5*0101 molecules in a recently developed biochemical assay.

**Conclusions/Significance:**

HLA-I binding 9mer influenza virus-derived peptides induce in many cases CD4^+^ T cell responses restricted by HLA-II molecules.

## Introduction

Influenza is a highly contagious, airborne respiratory tract infection associated with a significant disease burden during seasonal influenza outbreaks every year. In addition, the emergence of a new influenza subtype A (H5N1) [1], which can be directly, although rarely, transmitted from birds to humans, and especially the recent outbreak of swine-origin H1N1 virus which is transmitted to and spread among humans are potential or actual pandemic flu threats, respectively [2], [3]. Currently, vaccinations using inactivated or live-attenuated influenza virus preparation remain the primary method of prevention, both of which are dominated by the antibody-mediated immune responses to the highly variable surface glycoproteins, hemagglutinin (HA) and neuraminidase (NA). However, the virus escapes vaccine-induced neutralizing antibodies through constantly changing the composition of its surface antigens. This complicates the development of cross-protective immunity i.e. the ability to cover several different isolates; rather, influenza vaccines must regularly be updated to match existing seasonal epidemic flu isolates.

It is known that CD8^+^ cytotoxic T lymphocyte (CTL) responses play a major role in the control of primary influenza virus infection [4], [5]. In mice, CTLs against conserved epitopes contribute to protective immunity against influenza viruses of various subtypes [6], [7]. The use of CTL epitopes, especially conserved ones shared by multiple viral strains, and identification of HLA class I (HLA-I) binding immunogenic peptides, might therefore be basis for a robust vaccine strategy against emerging influenza epidemics.

We have previously performed a genome-, pathogen-, and HLA-wide search for conserved CTL epitopes derived from influenza A virus [8]. The predicted CTL epitopes were synthesized and tested by biochemical methods for binding to the appropriate recombinant HLA-I protein, and by IFNγ ELISPOT analyses for CTL immune responses using PBMC from healthy, adult and HLA-typed Danish subjects, assumed to have been exposed to multiple influenza infections during the past. Using these technologies we identified 10 new antigenic flu-derived peptide epitopes [8]. However, this search for *conserved* CTL epitopes skewed the selection towards peptides derived from polymerase and nucleoproteins, whereas the classical flu antibody targets, HA and NA, only included 8 of the 167 predicted CTL epitopes. Although the surface glycoproteins HA and NA are very variable over time, they might still contain pivotal CTL epitopes and our previous demands for conservation among a large number of viral strains might have missed important HA- and NA-derived CTL epitopes.

In our recent work on pox-derived epitopes [9], [10], we became aware that the measured immune responses of peripheral blood mononuclear cells (PBMC) *in vitro* by IFNγ ELISPOT towards high affinity HLA-I binding 9mer peptides, were not solely restricted by the HLA-I molecule of the peptide presenting cells. By the use of anti-CD4, anti-CD8, anti-HLA-I, and anti-HLA class II (HLA-II) blocking antibodies, and by performing CD4^+^ and CD8^+^ T cell depletion experiments on PBMC prior to ELISPOT expansion cultures, we demonstrated that T cells in the peripheral blood of vaccinia virus vaccinated and responding individuals gave rise to both the expected typical HLA-I restricted, CD8^+^ T cell dependent responses, as well as unexpected responses mediated by CD4^+^ T cells and apparently restricted by HLA-II [10].

In the present study, we have screened 9mer peptide epitopes from available influenza A viral protein sequences including the highly variable surface glycoproteins HA and NA by using the NetCTL algorithm for epitope prediction [11] and the EpiSelect algorithm for broad coverage of all available Influenza A strains [12]. In addition, we analyzed whether the predicted HLA-I binding 9mer peptides induced reactivity by CD4^+^ T cells. Twenty-one peptides of the 131 HLA-I binding peptides studied, were found to induce T cell responses in donors typed for the corresponding HLA-class I allele. However, only 5 peptides induced strictly CD8^+^ T cell dependent HLA-I restricted responses whereas 16 peptides induced CD4^+^ T cell dependent responses.

## Results

### Prediction of influenza A CTL epitopes

Bioinformatics (NetCTL) was used to identify broadly immunogenic influenza A-specific CTL epitopes restricted to one of the 12 HLA-I supertypes [13]. A number of predicted epitopes, that together constitute a broad coverage among different influenza A strains, were selected using the EpiSelect algorithm used previously to select HIV-1 epitopes [12]. In total, 146 predicted CTL epitopes were selected. In average, each predicted epitope is found in 442 different strains and Table 1 specifies this according to the protein in question. The influenza strains initially used for predicting epitopes mainly belong to five subtypes: H3N2, 124 epitope predicted (ep); H1N1, 68 ep; H5N1, 35 ep; H2N2, 55 ep; and H1N2, 86 ep. The Table shows that a predicted HA1 epitope is on average found in 35% of the sequenced HA1 proteins, while a predicted NP epitope is on average found in 84% of the sequenced NP proteins. These percentages reflect the level of protein conservation.

**Table 1:** Distribution of the 146 epitopes in different influenza A proteins, and coverage of the predicted epitopes against different strains of influenza A viruses.

Protein name	Predicted epitopes in percent of the number of sequenced proteins
Hemagglutinin 1 (HA1)	35%
Hemagglutinin 2 (HA2)	51%
Neuraminidase (NA)	47%
Nonstructural protein 1 (NS1*)*	8%
Nonstructural protein 2 (NS2)	69%
Matrix protein 1 (M1)	51%
Matrix protein 2 (M2)	69%
Nucleoprotein (NP)	84%

### Biochemical validation of HLA-I binding

131 of the 146 predicted HLA-I binding epitopes were synthesized (the remainder were rejected due to problems with synthesis or dissolving of the peptides). To determine whether the peptides indeed were binders to the relevant HLA-I proteins, they were tested for binding to each of the 12 HLA-A or –B supertypes in a biochemical assay (see Materials and Methods) (Table 2). Consistent with previous classifications, the binding affinity (*K*_D_) of the 131 predicted 9mer peptides can be divided into groups of high (*K*_D_≤50 nM) and intermediate affinity binders (50 nM<*K*_D_≤500 nM), respectively, as well as low affinity binders (500 nM<*K*_D_≤5000 nM), and peptides with no affinity (*K*_D_>5000 nM) for MHC-I molecules (four peptides). As shown in the Table 2, 87 of the 131 predicted peptides, or 66%, turned out to be high or intermediate affinity binders.

**Table 2:** Measured HLA-I/peptide affinity of the predicted peptide binders.

HLA supertype	K_D_ a ≤50^b^	50< K_D_ ≤500^b^	500< K_D_ ≤5000	K_D_ >5000	Total
**A1**	1	4	2	7	14
**A2**	6	1	1	0	8
**A3**	2	5	3	0	10
**A24**	5	3	0	1	9
**A26**	4	3	1	3	11
**B7**	4	3	1	5	13
**B8**	0	3	0	5	8
**B27**	3	6	3	1	13
**B39**	2	4	1	3	10
**B44**	7	6	0	1	14
**B58**	2	5	3	0	10
**B62**	2	6	0	3	11
**Total**	38	49	15	29	131

a K_D_, the equilibrium dissociation constant; a measurement of the affinity of peptides binding to the relevant HLA molecules in nM, the lower the value, the stronger the binding.

b High and intermediate peptide binding affinity.

### Immunogenicity of the predicted peptides

All 131 peptides were tested for their ability to stimulate influenza A-specific, HLA-matched T cells from a cohort of HLA-matched healthy Danish subjects aged 35–65, *ie.* assumed to have been exposed previously to natural influenza A virus. The peptides were evaluated for their ability to stimulate IFNγ production in an ELISPOT assay by PBMC from HLA-matched donors. In order to expand the frequency of peptide-specific T cells, PBMC were exposed for 10 days to peptides prior to performing the ELISPOT assays. Positive reactivity towards peptides was confirmed at least twice in the same donor as well as in other HLA supertype matched donors. IFNγ spot formation was detected for 21 peptides with binding affinity for one of the ten supertypes (A1, A2, A3, A24, A26, B7, B8, B44, B58, and B62). The ELISPOT data for these 21 antigenic peptides are shown in Table 3 and demonstrate that IFNγ spot-forming cell numbers per 10^5^ PBMC varied for the different peptides as well as between donors reactive for the same peptide (latter data not included). Table 3 also shows that some peptides were recognized by several donors whereas other peptides were only recognized by T cells from a single donor. Of note is that the four peptides (PF-110, PF-140, PF-146 and PF-148) without binding affinity for the predicted HLA supertypes HLA-A26, B62, B7 and B7, respectively, were recognized by PBMC of several donors in a HLA-II restricted manner.

**Table 3:** IFNγ ELISPOT analysis of peptide-specific donor responses.

Peptide #	Name	Sequence	HLA	K_D_ (nM)	Donors tested	Responding donor #	- Peptide a	+Peptide a
PF-96	NP_140–148_	HSNLNDTTY	A1	130	16	**32**	1±1	38±7
PF-103	NS1_128–136_	IMLKANFSV	A2	1	15	**1**	1±1	18±4
PF-116	NA_148–156_	TIHDRIPHR	A3	62	16	**29**	1±1	131±13
PF-106	M1_109–117_	FYGAKEIAL	A24	42	6	21, **25**	0±1	149±8
PF-109	HA_407–415_	KFHQIEKEF	A24	1	6	21, **25**	1±1	222±14
PF-110	HA_315–323_	VTIGECPKY	A26	20000	4	**25**	0±0	247±13
PF-113	NS1_142–150_	ETIVLLRAF	A26	28	4	**25**	0±0	159±17
PF-145	NA_281–289_	YPRYPGVRC	B7	11	17	21, **31**	11±5	106±12
PF-146	M2_24–32_	DPLVVAASI	B7	20000	17	**23**, 31	3±2	42±8
PF-147	HA_303–311_	LPFHNVHPL	B7	6	17	6, **9**, 10, 23, 31	3±1	210±11
PF-148	HA_544–552_	LVSLGAISF	B7	20000	17	6, 9, 10, 15, 21, **23**, 25, 31	6±2	157±13
PF-150	HA_307–315_	LPFQNVHPV	B7	161	17	8, **9**, 10, 23, 31, 32	2±2	139±10
PF-152	HA_324–332_	YVKQNTLKL	B7	72	17	**32**	7±9	46±5
PF-154	HA_266–274_	IAPWYAFAL	B8	7559	9	**14**, 19, 23	1±2	289±30
PF-156	M1_208–216_	QARRMVQAM	B8	87	9	**14**,19, 23	2±1	149±40
PF-130 b	NP_251–259_	AEIEDLIFL	B44	1	10	**2**, 9, 13, 27	6±3	85±9
PF-132	M1_7–15_	VETYVLSII	B44	13	10	1, 2, **9**	1±1	24±6
PF-135	NS1_179–187_	GVLIGGLEW	B58	553	5	35, **36**	1±1	51±9
PF-137	NS2_6–14_	VSSFQDILL	B58	687	5	**35**	2±1	11±3
PF-140	M2_39–47_	ILWILDRLF	B62	20000	6	**29**	1±1	17±4
PF-141	NA_159–167_	LMNELGVPF	B62	194	6	**17**	1±1	97±15

a Spot forming cell numbers represent an individual donor (in bold) and entries are the means of six individual assay cultures and in the absence or presence of peptide; differences are significant at P<0.05.

b Indicates already known epitope.

### Reactivity to the majority of peptides is blocked by a pan anti-HLA-II antibody

To ascertain whether, or not, CD4^+^ T cells are involved in the anti-influenza responses documented above, a pan-specific anti-HLA-II blocking antibody IVA12 as well as the anti-pan HLA-I antibody W6/32 were added into individual ELISPOT microcultures (see Materials and Methods). As shown in Table 4, reactivity towards 15 of the antigenic peptides, including the four HLA-I nonbinders, was fully inhibited by IVA12, but not, or only partially, by W6/32 antibody. Of the remaining 6 peptides reactivity against 5 peptides could be blocked by W6/32 but not by IVA12 antibody, whereas neither of the antibodies blocked reactivity against peptide PF137.

**Table 4:** Enzyme-linked immunospot (ELISPOT) responses against antigenic peptides in the absence or presence of HLA class I or HLA class II blocking antibodies.

					SFC/1×10^5^ PBMC+Peptide			
Peptide	Name	Sequence	HLA-I	KD (nM)	Donor#	Isotype	W6/32	IVA12
PF-96 a	NP_140–148_	HSNLNDTTY	A1	130	32	10±1	6±1* c	14±1
PF-103	NS1_128–136_	IMLKANFSV	A2	1	1	24±2	23±5	1±1 *
PF-116	NA_148–156_	TIHDRIPHR	A3	62	29	120±21	121±10	9±2 *
PF-106	M1_109–117_	FYGAKEIAL	A24	42	21	104±4	65±6 *	14±3 *
PF-109	HA_407–415_	KFHQIEKEF	A24	1	25	60±24	73±8	1±2 *
PF-110	HA_315–323_	VTIGECPKY	A26	20000	25	163±28	165±4	5±3 *
PF-113	NS1_142–150_	ETIVLLRAF	A26	28	25	96±6	105±16	2±1*
PF-145 a	NA_281–289_	YPRYPGVRC	B7	11	21	29±4	10±5*	39±7
PF-146	M2_24–32_	DPLVVAASI	B7	20000	31	17±1	18±3	2±2*
PF-147	HA_303–311_	LPFHNVHPL	B7	6	23	62±3	61±16	2±2 *
PF-148	HA_544–552_	LVSLGAISF	B7	20000	23	147±11	187±23	75±49 *
PF-150	HA_307–315_	LPFQNVHPV	B7	161	31	150±7	130±13	3±2 *
PF-152	HA_324–332_	YVKQNTLKL	B7	72	32	10±3	5±2	0±1 *
PF-154	HA_266–274_	IAPWYAFAL	B8	7559	23	38±15	38±10	1±1 *
PF-156	M1_208–216_	QARRMVQAM	B8	87	14	59±4	67±16	2±2 *
PF-130 a,b	NP_251–259_	AEIEDLIFL	B44	1	9	73±3	40±4 *	81±2
PF-132 a	M1_7–15_	VETYVLSII	B44	13	2	34±8	2±1 *	43±13
PF-135 a	NS1_179–187_	GVLIGGLEW	B58	553	36	30±7	4±4 *	26±10
PF-137	NS2_6–14_	VSSFQDILL	B58	687	35	18±4	16±1	19±4
PF-140	M2_39–47_	ILWILDRLF	B62	20000	29	32±3	43±19	1±1*
PF-141	NA_159–167_	LMNELGVPF	B62	194	17	152±21	66±21*	2±2 *

a Reactivity inhibited by anti-HLA class I mAb represents HLA-I restricted epitopes.

b Indicates already known epitope.

c *significant inhibition, *P*<0.05.

### Depletion of CD4^+^ and CD8^+^ T cells from PBMC

**Figure 1:**
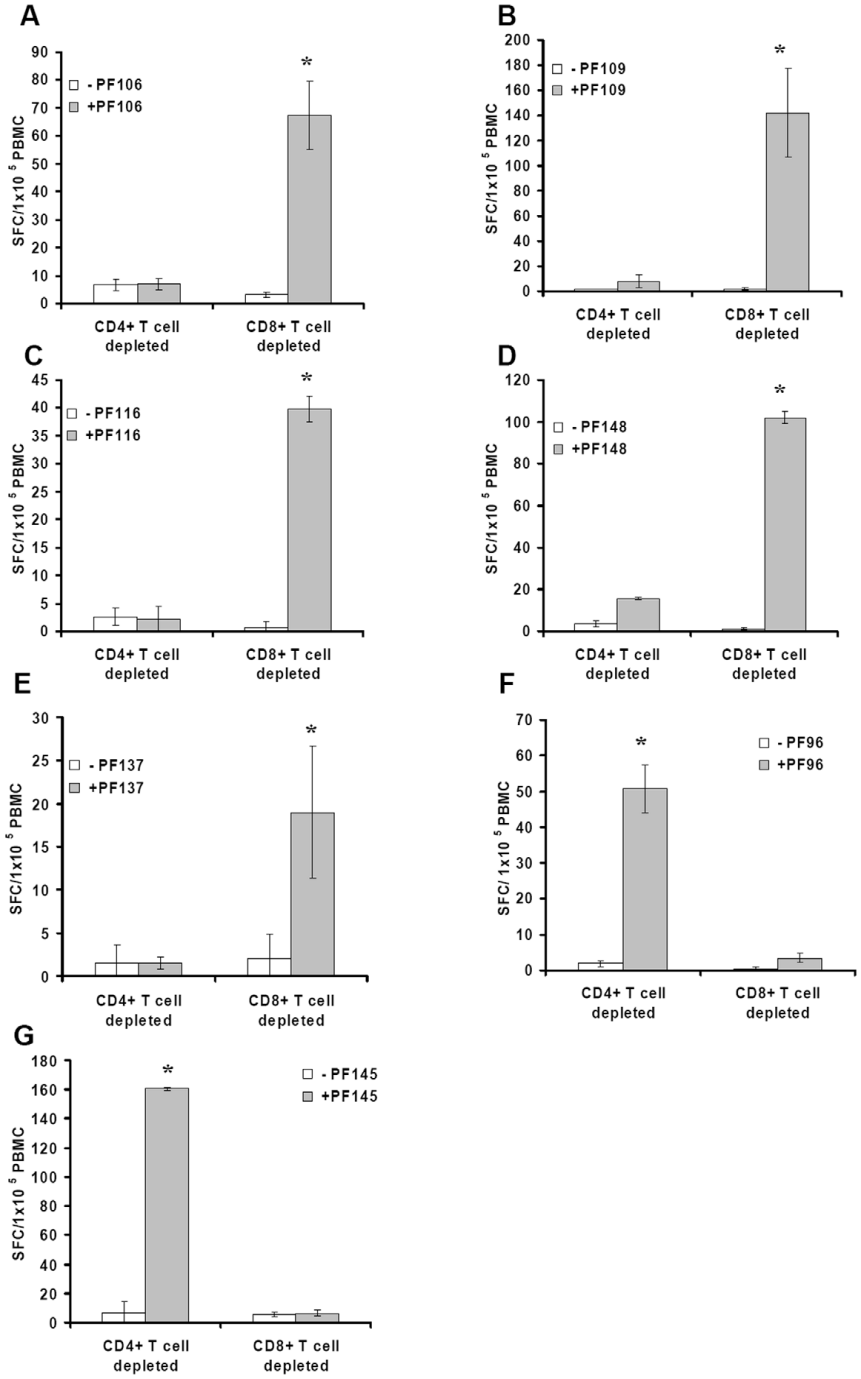
9mer flu-derived peptides induce CD4^+^ or CD8^+^ T cell responses. Peripheral blood mononuclear cells (PBMC) obtained from donors 21 (A), 25 (B), 29 (C) 23 (D), 35 (E), 32 (F) and 21 (G) were depleted of CD4^+^ T cells or CD8^+^ T cells and incubated with the indicated peptides for 10 days. Prior to testing, cells were harvested, washed and incubated in enzyme-linked immunospot (ELISPOT) plates for 20 h in the absence or presence of the indicated peptides. Results are expressed as the mean spot-forming cell values of four replicate ELISPOT microcultures, each containing 1×10^5^ CD4^+^ or CD8^+^ T cell-depleted PBMC. *, *P*<0.05 (Student's t- test).

To obtain direct evidence for the CD4 or CD8 phenotype of the responding T cells depicted in Table 4, T cell reactivity was tested on a number of selected peptides (Fig. 1) for which the reactivity was blocked by either anti-MHC-I or MHC-II antibodies (Table 4). PBMC were depleted for either CD4^+^ or CD8^+^ T cells prior to exposure to peptides. Figures 1A–E show reactivity obtained from PBMC of donor 21, 25, 29, 23 and 35 induced by peptides PF106, PF109, PF116, PF148 and PF137, respectively. It is evident that PBMC depleted of CD8^+^ T cells respond significantly in ELISPOT culture, whereas the CD4^+^ T cell-depleted PBMC do not respond at all. These results thus support the data in Table 4, although in some cases, the presence of W6/32 partially blocks reactivity (see Table 4 and discussion). Together, the data above indicate, that the CD4^+^ T cell- responses are restricted by HLA-II molecules. Despite not being blocked by the W6/32 or IVA12 antibodies (see Table 4), peptide PF137 induces a CD4^+^ T cell response (Fig. 1E). Figure 1F and G show that depletion of CD8^+^ T cells totally removed the responses against peptides PF96 and PF145 whereas depletion of CD4^+^ T cells did not inhibit responses in the ELISPOT culture thus confirming the blocking by W6/32 antibody (Table 4) of these responses. Collectively, the T cell mediated reactivity against the 21 antigenic 9mer flu-derived peptides studied here suggest that the peptides are recognized either by CD4^+^ or CD8^+^ T cells but not by both cell subsets. Thus, the two sets of responses appear to be mutually independent.

### HLA-II subtype specific blocking of peptide reactivity

**Figure 2:**
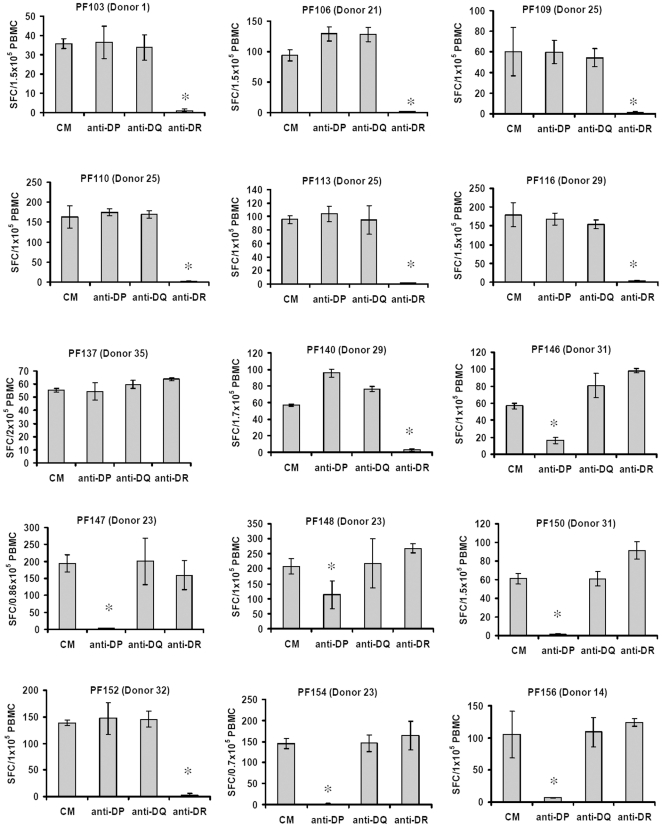
Flu-derived 9mer peptides induce HLA-DR- or -DP restricted CD4^+^ T cell responses. PBMCs from responding donors were incubated with the indicated peptides for 10 days. Prior to testing, cells were harvested, washed and exposed to the peptides in ELISPOT plates for 20 h in the absence or presence of anti-HLA-DP, DQ or DR mAbs. Results are expressed as the mean SFC numbers (± standard deviation) of four replicate ELISPOT microcultures. Bars represent standard deviation; *significant inhibition, P<0.05 (Student's t- test).

The peptide responses blocked by an anti-pan HLA-II antibody (IVA12) (Table 4) were further analyzed using HLA-II subtype specific antibodies for blocking of IFNγ spot formation. PF-141 was not studied due to loss of donor 17 PBMC. Fig. 2 shows the results. Eight individual peptide reactivities were totally blocked in the presence of anti-HLA-DR antibody and six peptide reactivities were blocked in the presence of anti-HLA-DP antibody whereas the presence of anti- HLA-DQ antibody was without any effect. As observed with the IVA12 antibody, the reactivity against peptide PF137 was not inhibited by either of the antibodies, although the separation experiment in Fig. 1E clearly shows that the reactivity is mediated by CD4^+^ T cells.

### Donor HLA-II subtypes and peptide binding to HLA-DR molecules

Donors giving rise to responses blocked by anti-HLA-I and –II antibodies were typed for HLA-A, B and DR, DQ and DP B loci expression by DNA sequencing (see Materials and Methods). Table 5 shows the typing data and the number of peptide epitopes recognized by individual donors.

**Table 5:** HLA class I and II subtypes for donors used in the present study.

			Sequence-based HLA-I typing				Sequence-based HLA-II typing								
Donor#	Sex	Age (y)	HLA-A		HLA-B		DRB1	DRB1	DRB345	DRB	DQB1	DQB1	DPB1	DPB1	EPI a
1	M	36	*0201	*0301	*3501	*4402	*0101	*1101	3*0202	-	*0301	*0501	*0401	-	2
2	M	47	*0101	*0301	*4901	*4001	*0301	*1301	3*0101	-	*0201	*0603	*0201	*1001	2
6	M	41	*0101	*0301	*0801	*3501	*0101	*0301	3*0101	-	*0201	*0501	*0401	*0101	2
9	F	43	*0301	*3201	*0702	*4402	*0801	*1501	5*0101	-	*0402	*0602	*0401	-	5
10	F	38	*0301	*2501	*0702	*1801	*0401	*1501	4*0103	5*0101	*0302	*0602	*0401	-	3
13	M	60	*0201	*0201	*3901	*4402	*0701	*1501	4*0103	5*0101	*0303	*0602	*0401	-	1
14	M	58	*0101	*0201	*0801	*3701	*0301	*0901	3*0101	4*0103	*0201	*0303	*0401	-	2
15	M	44	*0201	*2601	*0702	*1401	*0701	*1301	3*0101	4*0101	*0202	*0603	*0401	*0402	1
17	F	35	*0201	*2902	*4403	*1501	nd^b^	nd	nd	nd	nd	nd	nd	nd	1
19	F	46	*0101	*0201	*0801	*4001	*0301	*1302	3*0101	3*0301	*0201	*0604	*0401	-	2
21	M	54	*0101	*2402	*0702	*3901	*1101	*1501	3*0202	5*0101	*0301	*0602	*0402	*0901	4
23	F	58	*0101	*2402	*0702	*0801	*0101	*0301	3*0101	-	*0201	*0501	*0401	-	6
25	F	62	*2402	*2601	*0702	*3801	*0301	*1501	3*0202	5*0101	*0201	*0602	*0201	*0402	5
27	F	43	*0201	*6801	*5701	*4402	*0101	*0701	4*0103	-	*0303	*0501	*0401	*0402	1
29	M	52	*0301	*1101	*1302	*1302	*0701	*1302	3*0301	4*0103	*0202	*0604	*0401	*0402	2
31	F	63	*0201	*0301	*0701	*5601	*1101	*1501	3*0202	5*0101	*0301	*0602	*0402	*0901	5
32	M	55	*0201	*3201	*1501	*5101	*0401	*1101	3*0202	4*0103	*0301	*0302	*0401	-	3
35	F	43	*2902	*3303	*5801	*2705	*0101	*0301	3*0202	-	*0201	*0501	*0401	*0402	2
36	F	50	*0201	*2402	*5701	*3508	*0101	*0701	4*0103	-	*0303	*0501	*0401	-	1

a number of epitopes recognized by the donor.

b nd, not done.

A high-throughput HLA-II peptide binding assay, recently developed in our laboratories, allows the affinities of HLA-II/peptide interaction to be determined in the nanomolar range [14]. The 14 peptides recognized by CD4^+^ T cells (Fig. 1) and for which reactivity was blocked by anti-DR or anti-DP antibodies (Fig. 2), respectively, were tested for binding to recombinant HLA-DRB1*0101, -DRB1*0301, -DRB1*0302, -DRB1*0401, -DRB3*0301, -DRB5*0101 chains combined with the non polymorphic DRA1*0101 chain and for binding to DPA1*0103/DPB1*0401 molecules. Table 6 shows the results. As positive controls for binding, two pan HLA-DR/DP binding 13 and 15mer peptides were included. Only those peptides showing a binding affinity <5000 nM are included in Table 6. Three of the 8 peptides (IMLKANFSV, ETIVLLRAF, YVKQNTLKL), for which reactivity was blocked by anti-DR antibody, showed binding to DRA1*0101/DRB1*0401 or DRA1*0101/DRB5*0101 also expressed by the reactive donors, whereas none of the peptides, for which reactivity was blocked by anti-DP antibody, showed binding to DPA1*0103/DPB1*0401, however, two of these peptides showed binding to DR molecules expressed by the reactive donors.

**Table 6:** Affinities of peptides to MHC-II alleles.

	Peptide sequence	DRB1*0101	DRB1*0301	DRB1*0302	DRB1*0401	DRB3*0301	DRB5*0101	DPA1*0103/DPB1*0401
**HA_306–318_**	**YKYVKQNTLKLAT**	108 a	26	53	15	3	12	NB
**hypothetical protein 239–253** [27]	**YILLKKILSSRFNQM**	435	20	156	107	3	17	25
DR/Donor 1 b	IMLKANFSV (PF-103)	3433 c	NB	NB	1387	18	NB	NB c
DR/Donor 25	ETIVLLRAF (PF-113)	3729	NB c	NB	NB	NB	780 c	NB
DP/Donor 23	LVSLGAISF (PF-148)	4340 c	NB^c^	NB	NB	NB	NB	NB c
DR/Donor 32	YVKQNTLKL (PF-152)	1203	NB	2221	226 c	356	405	NB c
DP/Donor 23	IAPWYAFAL (PF-154)	NB c	NB c	NB	NB	NB	1696	NB c
Donor 17 d	LMNELGVPF (PF-141)	NB	NB	NB	NB	4014	NB	NB
DP/Donor 23	LPFHNVHPL (PF-147)	NB c	NB c	NB	NB	NB	3704	NB c
DP/Donor 31	LPFQNVHPV (PF-150)	NB	NB	NB	NB	NB	1204 c	NB c

Note: peptides marked in bold are positive controls included in the assay; HA_306–318_ is a promiscuous HLA-DR binder whereas hypothetical protein 239–253 has been shown to bind most HLA-DR alleles and the HLA-DP allele. Peptide showing affinities above 5000 nM are categorized as non-binders (NB).

a Peptide binding affinity (K_D_) in nM.

b Peptide reactivity blocked by anti-HLA-II subtype antibody in the responding donor number (See Fig. 2 and Table 5).

c The HLA-II subtype assayed for peptide binding was expressed by donor.

d No cells available for HLA-II typing.

## Discussion

Previously, we have performed a genome-, pathogen-, and HLA-wide search for conserved CTL epitopes in influenza A virus [8]. However, this search for *conserved* CTL epitopes skewed the selection towards the polymerase and nucleoprotein, whereas the classical antibody targets, HA and NA, were found to contain only a few (8) of the 167 predicted CTL epitopes [8]. Instead of searching for conserved CTL epitopes, we attempted in the present study, to select a number of predicted HLA-I binding influenza A CTL epitopes, which constitute a broad coverage of all available influenza A strains. According to this criterion, most of the predicted CTL epitopes from the PB1, PB2, and PA proteins were found to be shared with those tested in our previous publication [8], and for the purpose of novelty, these proteins were therefore excluded from the present study.

To increase the chance for the discovery of new peptide epitopes, we tested the peptides in all HLA-I supertype matched donors available to us. In the present work, we discovered 20 new immunogenic peptides, and confirmed one known, of 131 peptides tested as compared to the discovery of 10 new and 3 known peptides of 167 flu peptides tested in our previous report [8]. In the latter case, the peptides were only tested in a few of the available HLA-matched donors.

In recent work on pox-derived epitopes, we found that immune responses of donor PBMC *in vitro*, as measured by IFNγ ELISPOT towards HLA-I binding 9 mer peptides, were either CD8^+^ or CD4^+^ T cell-dependent and that the latter appeared to be restricted by HLA-II molecules [10]. This observation led us to investigate whether the predicted HLA-I binding flu peptides of the present study induce CD4^+^ T cell-dependent responses. The key finding of the present study is that 16 of 131 9mer peptides derived from influenza A viral proteins induce CD4^+^ T cell dependent responses in vitro from presumably immune donors. These responses were, with one exception, all blocked by anti-HLA-II antibody. In addition, 8 peptide responses were blocked by an anti-HLA-DR antibody and 6 peptide responses by an anti-HLA-DP antibody. Surprisingly, only 5 peptide responses (including the known peptide PF130 [15], [16]), were blocked by a HLA-I antibody. For selected peptides, CD4^+^ and CD8^+^ T cell depletion experiments showed that CD4^+^ T cell responses were blocked by anti-HLA-II antibody, whereas CD8^+^ T cells were blocked by anti-HLA-I antibody. As shown in Table 4, the anti-HLA-I antibody in fact showed partially blocking of reactivity for some peptide epitopes (PF106 and PF141) that, according to the cell depletion experiments (Fig. 1) induced CD4^+^ T cell, but not CD8^+^ T cell, responses. Such partial blocking might reflect anti-HLA-I antibody-mediated apoptosis of activated effector CD4^+^ T cells as previously demonstrated [17], [18]. The fact that CD4^+^ T cell-mediated responses against PF137 is not inhibited by any of the two anti-HLA antibodies might suggest an excessively high stimulatory binding avidity of peptide specific CD4^+^ T cells.

It is generally accepted that HLA class I binding peptides are composed of 8-11 amino acids, whereas HLA class II binding peptides consist of 15–20 amino acids being recognized by CD8^+^ and CD4^+^ T cells respectively [19]–[21]. Both HLA-I and -II molecules bind to primary and secondary peptide anchor motifs covering the central 9-10 amino acids. Thus, considering this common structural basis for peptide binding to HLA-I and –II molecules, the present finding of 9mer peptide binding to HLA-II molecules is not unexpected (also documented in the immune epitope database www.Immuneepitope.org). As mentioned above, we have previously reported that high-affinity HLA-I binding variola-derived 9mer peptides (K_D_ <6 nM) induce CD4^+^ T cell responses ex vivo more than 30 years post-vaccinia virus vaccination, which can be blocked by anti-HLA-II antibody [10]. The same phenomenon was observed here: *in silico* predicted HLA-I binding 9mer peptides – this time derived from influenza A viral proteins - induce CD4^+^ T cell mediated responses which appear to be HLA-II restricted as T cell responses are totally blocked by a pan HLA-II antibody. In contrast to our previous study [10], we found no correlation between CD4^+^ T cell reactivity and HLA-I binding affinity of peptides. The induction of immune responses in the present study was not limited to high-affinity HLA-I binding peptides. Rather, we found examples of peptides with intermediate, low or no binding affinities for its HLA-I allele. Such peptides were all capable of stimulating strong CD4^+^ T cell responses, again suggesting that these 9mer peptides are presented by HLA-II molecules. As indicated from data in Table 3 and 4 only half of the HLA-II restricted responses were observed in more than one donor whereas four of the five HLA-I restricted responses were observed in at least two donors. This difference might reflect the relatively low binding affinity of 9mer peptides for HLA-II (Table 6) as opposed to their binding affinity for HLA-I (Table 4), thereby making the ELISPOT assay less sensitive as a readout for antigenic HLA-II binding 9mer peptides.

Using a high-throughput HLA-II peptide binding assay, recently developed in our laboratories [14], we found that 3 of 8 peptides, for which reactivity was blocked by anti-DR antibody, bind to donor HLA-DR subtypes. These numbers are higher than expected since only one quarter of the HLA-DR alleles expressed by the peptide reactive donors was assayed for peptide binding. Regarding the peptides, for which reactivity was blocked by anti-DP antibody, it was quite surprising to find that none of these peptides bind to DPA1*0103/DPB1*0401 although the majority of donors in our material express this common HLA-II subtype. At present we have no explanation for this negative observation.

Frahm et al. [22] have recently tried to determine the HLA class I promiscuity of previously well-defined CTL epitopes by testing responses in 100 subjects to a set of 242 HIV and EBV-derived CTL epitopes using PBMC in IFNγ ELISPOT assays, regardless of the individual's HLA type. Fifty percent of all positive responses were detected in individuals who did not express originally described restricting HLA-I allele. The authors concluded that epitope presentation and CTL recognition might occur frequently in the context of alternative HLA class I alleles. Although some alternative HLA-I restrictions were confirmed experimentally, the majority were not identified, but inferred by statistical methods [22]. Our previous [10] and present results showing HLA-I binding 9mer peptides capable of activating CD4^+^ T cell dependent responses, may suggest that some of the “alternative restricted” responses described by Frahm et al. [22] reflect CD4^+^ T cell recognition of epitopes restricted by HLA-II.

Assarsson et al [23] have used the similar strategy as ours to identify influenza A virus-derived epitopes, but none of their discovered epitopes matched those discovered in our study. The discrepancy between the two studies might be explained by the fact that Assarsson et al mainly focused on epitopes derived from conserved sequences while we focused on epitopes in influenza virus regardless of their conservancy. Also they used freshly harvested PBMC whereas we used in vitro restimulated PBMC which increases the frequency of influenza virus-specific T cells thereby enhancing the detection sensitivity. Indeed, for some antigenic peptides in [23], the numbers of SFC in the ELISPOT assay are very low. In line with this, some epitopes including two HLA-I restricted (PF-96 and PF-132) and five HLA-II restricted epitopes (PF-103, PF-109, PF-146, PF-152 and PF-PF-141) identified in our study, were also included in [23] but failed to show antigenicity. Furthermore, Assarsson et al only discovered 54 epitopes out of 4080 peptides tested (discovery rate: 1.3%), while we identified 21 out of 131 peptides (discovery rate: 16%) by using peptide restimulated T cells. Therefore, the in vitro restimulation might be needed in PBMC of unvaccinated individuals prior to performance of ELISPOT assays in order to increase the detection sensitivity. Intriguingly, Assarsson et al. did not discover any HLA-II restricted epitopes among 38 HLA-I binding epitopes (8–11mer), whereas we identified 16 of 21 HLA-I binding 9mer peptides in our study as HLA-II restricted CD4^+^ T cell epitopes. However, they excluded some 8–11mer peptides which induced reproducible positive responses, but showed poor binding ability to the relevant HLA-I alleles. It would be interesting to know if the responses induced by these latter peptides are CD4^+^ T cell dependent.

We propose that studies, which employ ‘reverse immunology’ to monitor HLA class I responses against HLA-I binding peptides by use of IFNγ ELISPOT assay, should take class II-restricted, CD4-dependent T cell responses into account. Our present and previous data [10] suggest that HLA-I binding peptides might stimulate CD4^+^ T cell immune responses restricted by HLA-II molecules. Thus, ELISPOT-based analyses of reactivity against 9mer class I binding peptides should always include either anti-CD4/CD8 blocking or CD4/CD8 T cell subset depletion experiments or, alternatively, perforin- or granzyme B-based ELISPOT analyses to obtain the true phenotype of the antigen-specific T cells.

In the present and previous [8] studies we have identified a total of 30 new antigenic flu-derived 9mer peptides (large proteins PB1,PB2,PA,NP,NA,HA and small proteins M1, M2, NS1) potentially recognized by the majority of humans disregarding their HLA allotype and group. We now plan to initiate animal vaccine studies in flu infected HLA transgenic mice to assay for the protective/therapeutic efficacy of the peptides. If a clinical effect is obtained, the peptides might be of use as vaccine candidates in future influenza pandemics.

In conclusion, by the use of PBMC from healthy adult donors, twenty one 9mer peptides derived from influenza A viral proteins were found to induce T cell responses in an IFNγ ELISPOT assay. Only 5 of the peptides induced HLA-I restricted CD8^+^ T cell responses. The remaining 16 peptides, of which 3 peptides were shown to bind to HLA-DR, induced CD4^+^ T cell responses apparently restricted by HLA- II molecules.

## Materials and Methods

### Collection of blood samples and tissue typing

Buffy coats of 500 ml whole blood from individuals in the Danish blood donor corps (age range: 35–65 years; including informed consent) were obtained from The Blood Bank at Rigshospitalet (Copenhagen, Denmark) and used within 24 hours to isolate peripheral blood mononuclear cells (PBMC). The donors were selected, according to serological typing of their HLA-A and -B haplotypes, to maximize coverage of the 12 HLA-I supertypes, including HLA-A1, -A2, -A3, -A24, -A26, -B7, -B8, -B27, -B39, -B44, -B58 and -B62 [13]. A high-resolution sequence-based typing (SBT) of the HLA-A, HLA-B and the HLA-DR,-DQ and-DP loci was subsequently established (Genome Diagnostics, Utrecht, Netherlands). Typing data are shown in Table 5.

This study was in accordance with the ethical guidelines, and approved by the Institutional Review Board, University Hospital of Copenhagen (Rigshospitalet), Denmark.

### Isolation of PBMC

Peripheral blood mononuclear cells (PBMC) were isolated from buffy coats by density gradient centrifugation using Lymphoprep (Nycomed Pharma AS, Oslo, Norway). The freshly isolated PBMC were cryopreserved for later use at 20×10^6^ cells in 1 ml RPMI-1640 containing 20% FCS and 10% dimethyl sulfoxide (DMSO) at −140°C.

### Bioinformatics search strategy for CTL epitopes derived from influenza A virus

The CTL epitope predictions were performed on the basis of a dataset consisting of 3735 influenza A strains obtained from the Influenza Sequence Database (www.flu.lanl.gov). The 3735 strains comprise a total of 10497 sequenced proteins of which the PB1, PB2, and PA proteins were excluded, since we have previously already tested 37 PB1-, 57 PB2-, and 39 PA-derived predicted CTL epitopes [8]. CTL epitopes derived from the remaining eight influenza proteins (HA1, HA2, NA, NS1, NS2, M1, M2, and NP) and restricted to any of the 12 HLA-I supertypes were predicted using the NetCTL 1.0 method [11] (available at www.cbs.dtu.dk/services/NetCTL). The NetCTL method uses a combined prediction score for MHC class I affinity, TAP transport efficiency, and C-terminal proteasomal cleavage as a weighted sum of the three individual prediction scores. For MHC class I affinity, the NetMHC-3,0 method is used. For TAP transport efficiency, the method of Peters et al. [24] is used, and for proteasomal cleavage, the NetChop C-term 3.0 method is used. Thus, in the NetCTL method, each possible 9mer in a protein is assigned a score based on a combination of proteasomal cleavage, TAP transport efficiency, and HLA-I binding affinity with the highest weight assigned to the HLA-I affinity. Depending on the size of the protein, between one and two of the top-scoring 9mers in each protein were selected as the predicted epitopes for each of the 12 HLA-I supertypes.

Next, the EpiSelect algorithm was used for selecting a number of predicted CTL epitopes, which together constitute a broad coverage of all strains. The EpiSelect algorithm has been described in detail previously [12]. The input to the algorithm is a set of lists defining the predicted epitopes in each viral strain. Briefly, the algorithm aims at selecting a given number of epitopes in a way so that the number of epitopes in the viral strain with fewest epitopes is as high as possible while simultaneously maximizing average coverage of all strains.

Initially, 168 CTL epitopes were selected corresponding to 14 epitopes per supertype. Twelve epitopes had been tested previously [8] and they were therefore excluded in the present work. Of the remaining 156 epitopes, 10 were restricted to two different HLA-I supertypes leaving us with a total of 146 predicted epitopes.

### Peptides

The 9mer peptides were synthesized by standard 9-fluorenylmethyloxycarbonyl (FMOC) chemistry, and purified by reversed-phase high-performance liquid chromatography (at least 80%, usually >95% purity) and validated by mass spectrometry (Shafer-N, Copenhagen, Denmark). Peptides were distributed at 500 µg/vial and stored lyophilized at −20°C until use. Peptides were dissolved just before use.

### Biochemical peptide-HLA-I and –II binding assays

The biochemical assay for peptide–MHC-I binding was performed as previously described [25]. Briefly, denatured and purified recombinant HLA heavy chains were diluted into a renaturation buffer containing β_2_-microglobulin and graded concentrations of the test peptide, and incubated at 18°C for 48 h allowing equilibrium to be reached. We have previously demonstrated that denatured HLA molecules can *de novo* fold efficiently, however, only in the presence of appropriate peptide [26]. The concentration of peptide–HLA complexes generated was measured using Luminescent Oxygen Channeling Immunoassay (LOCI) and plotted against the concentration of peptide offered. Because the effective concentration of HLA (1–3 nM) used in these assays is below the equilibrium dissociation constant (K_D_) of most high-affinity peptide–HLA interactions, the peptide concentration leading to half-saturation of the HLA is a reasonable approximation of the affinity of the interaction.

Affinity measurements of peptides to recombinant HLA-DRB1*0101,-DRB1*0301, -DRB1*0302, -DRB1*0401, -DRB3*0301, -DRB5*0101 and DPA1*0103/DPB1*0401 molecules were done according to previous work [14]. Briefly, peptides including reference peptides knowing to bind the used HLA-II alleles [DR-binding peptide HA 308-318 (sequence: YKYVKQNTLKLAT) and plasmodium falciparum 3D7 derived DP-binding peptide, hypothetical protein 239-253 [27] (sequence: YILLKKILSSRFNQM)] were dissolved and titrated in 25% glycerol, 0.1% pluriol (F68), 150 mM NaCl. A HLA-II stock solution consisting of bacterially expressed and urea denatured alpha and beta chains, at appropriate concentrations were diluted into refolding buffer: 100 mM Tris/Citrate, 25% Glycerol 0.01% Pluriol F68 containing protease inhibitors (TPCK and Pepstatin both 3,3 µg/ml) at pH 6 (DRB1*0101. DRB5*0101) or 7 (remaining HLA-II alleles). The diluted HLA-II stock was subsequently mixed 1∶1 with peptide titrations and incubated at 18°C for 48 hrs. Formed HLA-II complexes were detected using a homogenous proximity assay (Alpha Screen, Perkin Elmer), briefly streptavidin coated donor beads and L243 (murine monoclonal anti DR) coupled acceptor beads, both 5 mg/ml, were diluted 500 times into PBS 0.1% Pluriol (F68). 10 µl of bead mix was mixed with 10 µl HLA- II/peptide samples in 384 Optiplates (Perkin Elmer). Following 18 hrs of incubation at 18°C they were read on an Envision Reader (Perkin Elmer) and analyzed according to [14].

### Depletion of CD4^+^ or CD8^+^ T cells from PBMC

CD4^+^ T cells or CD8^+^ T cells were positively depleted from PBMC according to the manufacturer's instruction using monoclonal anti-CD4-coated or monoclonal anti-CD8-coated Dynabeads from Dynal Biotech ASA (Oslo, Norway). PBMC depleted of CD4^+^ T or CD8^+^ T cells were verified by flow cytometry.

### IFNγ ELISPOT assay

The PBMC were thawed, washed and then used for CD4^+^ or CD8^+^ T cell depletion (see Materials and methods) or cultured directly in RPMI-1640 supplemented with 5% heat-inactivated AB serum (Valley Biomedical, Winchester, VA, USA), 2 mM L-glutamine, 100 U/ml penicillin and 100 µg/ml streptomycin. PBMC (4–6×10^6^) or depleted PBMC were cultured in 1 ml culture medium in 24-well plates (Nunc, Roskilde, Denmark) in the presence of individual peptides with a final concentration of 10 µg/ml per well, and incubated for 10 days at 37°C, 5% CO_2_ in humidified air. Recombinant human (rh) IL-2 (Proleukin; Chiron, Amsterdam, the Netherlands) 20 U/ml was added on day 1. Cells were harvested on day 10, washed twice in RPMI-1640 and resuspended in complete medium to a final concentration of 1–2×10^6^ cells/ml. The IFNγ ELISPOT assay was performed to quantify peptide-specific T cells after *in vitro* expansion as described previously [8]. In brief, the expanded PBMC, 1–2×10^5^, were cultured for 20 hours in the presence (six wells) or absence (six wells) of indicated peptides with a final concentration of 10 µg/ml in an ELISPOT plate. As positive controls, cells were stimulated with 10 µg/ml phytohaemagglutinin (Sigma-Aldrich, Poole, Dorset, UK). Attempts to block HLA-I and HLA-II restricted responses were performed in 4 cultures of 10 days expanded cells. To block HLA-I-restricted responses the pan specific anti-HLA-I antibody W6/32 ascites (ATCC) was added at a final dilution of 1∶40 for 30 min before adding peptides in ELISPOT assays. To block pan HLA-II-restricted responses, 10 µg/ml anti-pan HLA-II monoclonal antibody IVA12 (ATCC, Rockville, MD, USA) was added; to block HLA-II subtype specific responses, 10 µg/ml of anti-DR (L243, ATCC), anti-DQ (SPV-L3, IgG2a, a kind gift from Dr. H.Spits, DNAX,CA,USA) and anti-DP (B7/21, Abcam,USA.) specific antibodies were added. In selected cases, PBMC depleted of CD4^+^ or CD8^+^ T cells prior to the 10 days expansion culture, were cultured in the presence or absence of indicated peptides in ELISPOT plates to confirm the dependence of T cell subsets responsible for peptide-induced responses. Data were expressed as the mean spot-forming cells (SFC) of 4 replicate assay cultures.

### Statistics

Student's *t*-test was used to analyze the quantitative differences between the experimental and control wells in ELISPOT assays. All tests were one-tailed and a P-value below 0.05 was considered significant.
